# Battery Separators Functionalized with Edge-Rich MoS_2_/C Hollow Microspheres for the Uniform Deposition of Li_2_S in High-Performance Lithium–Sulfur Batteries

**DOI:** 10.1007/s40820-019-0275-z

**Published:** 2019-05-24

**Authors:** Nan Zheng, Guangyu Jiang, Xiao Chen, Jiayi Mao, Nan Jiang, Yongsheng Li

**Affiliations:** Lab of Low-Dimensional Materials Chemistry, Key Laboratory for Ultrafine Materials of Ministry of Education, Shanghai Engineering Research Center of Hierarchical Nanomaterials, School of Materials Science and Engineering, East China University of Science and Technology, Shanghai, 200237 People’s Republic of China

**Keywords:** Edge-rich, MoS_2_/C, Hollow microspheres, Li_2_S, Lithium–sulfur batteries

## Abstract

As promising energy storage systems, lithium–sulfur (Li–S) batteries have attracted significant attention because of their ultra-high energy densities. However, Li–S battery suffers problems related to the complex phase conversion that occurs during the charge–discharge process, particularly the deposition of solid Li_2_S from the liquid-phase polysulfides, which greatly limits its practical application. In this paper, edge-rich MoS_2_/C hollow microspheres (Edg-MoS_2_/C HMs) were designed and used to functionalize separator for Li–S battery, resulting in the uniform deposition of Li_2_S. The microspheres were fabricated through the facile hydrothermal treatment of MoO_3_–aniline nanowires and a subsequent carbonization process. The obtained Edg-MoS_2_/C HMs have a strong chemical absorption capability and high density of Li_2_S binding sites, and exhibit excellent electrocatalytic performance and can effectively hinder the polysulfide shuttle effect and guide the uniform nucleation and growth of Li_2_S. Furthermore, we demonstrate that the Edg-MoS_2_/C HMs can effectively regulate the deposition of Li_2_S and significantly improve the reversibility of the phase conversion of the active sulfur species, especially at high sulfur loadings and high C-rates. As a result, a cell containing a separator functionalized with Edg-MoS_2_/C HMs exhibited an initial discharge capacity of 935 mAh g^−1^ at 1.0 C and maintained a capacity of 494 mAh g^−1^ after 1000 cycles with a sulfur loading of 1.7 mg cm^−2^. Impressively, at a high sulfur loading of 6.1 mg cm^−2^ and high rate of 0.5 C, the cell still delivered a high reversible discharge capacity of 478 mAh g^−1^ after 300 cycles. This work provides fresh insights into energy storage systems related to complex phase conversions.

**Electronic supplementary material:**

The online version of this article (10.1007/s40820-019-0275-z) contains supplementary material, which is available to authorized users.

## Highlights

- Edge-rich MoS_2_/C hollow microspheres (Edg-MoS_2_/C HMs) were fabricated through the simple hydrothermal treatment of MoO_3_–aniline nanowires and a subsequent carbonization process.
- The Edg-MoS_2_/C HMs ensure the uniform deposition of Li_2_S on the matrix and the enhanced utilization of active edge sites.
- The cell with an Edg-MoS_2_/C HM-functionalized separator displayed excellent electrochemical performance, with a high reversible discharge capacity of 478 mAh g^−1^ after 300 cycles at a high sulfur loading of 6.1 mg cm^−2^ and high rate of 0.5 C.

**Electronic supplementary material:**

The online version of this article (10.1007/s40820-019-0275-z) contains supplementary material, which is available to authorized users.

## Graphical abstract

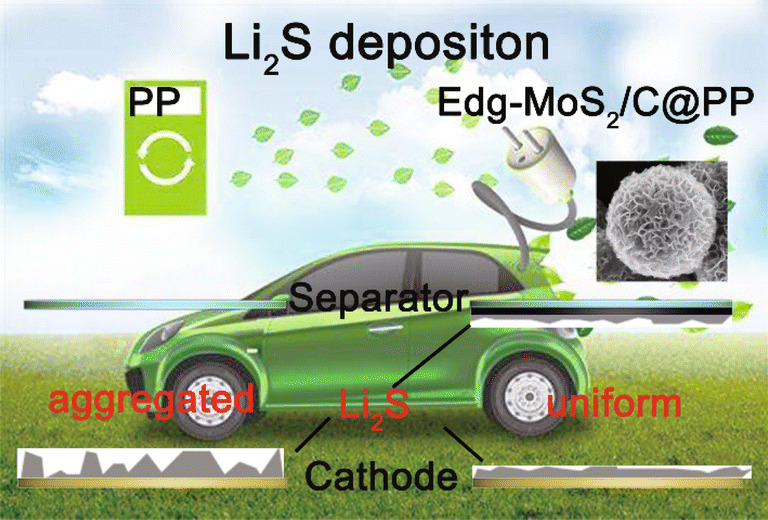

## Introduction

Because of their high cost and limited capacities, conventional lithium-ion batteries cannot meet the constantly increasing demands of portable electronic devices and electrified transportation [1–5]. Therefore, the development of advanced battery systems with high energy densities, superior rate performances, and long cycle stabilities is increasingly urgent. Lithium–sulfur (Li–S) batteries have attracted intense interest because of their ultra-high energy densities (2600 Wh kg^−1^) and the low cost of the active material (sulfur), as well as its high natural abundance and environmental friendliness [6–8]. Despite these tremendous merits, the further development of Li–S batteries is hindered by the problems resulting from the complex phase conversion processes that occur during battery charging and discharging [7, 9–11].

In the first stage of the discharge process in Li–S batteries, solid S is converted into soluble polysulfides (Li_2_S_*x*_, 4 ≤ *x* ≤ 8) in the cathode [7]. Because of osmosis, the permeation of polysulfides through the separators to the Li metal anodes occurs easily, leading to the loss of active sulfur and the corrosion of the Li metal anodes [7]. Thus, the reversible capacities of Li–S batteries fade fast and “terrible” lithium dendrites form [2]. Various strategies have been devoted to blocking the diffusion of polysulfides, thus improving the electrochemical performance of Li–S batteries, including sulfur host design [6, 12–18], separator functionalization [6, 19–28], and new electrolyte exploration [29–31]. Of these methods, separator functionalization is a promising strategy to restrict the shuttling of the polysulfides and increase the utilization of active sulfur. The materials used to functionalize separators can be divided into two main categories: first, carbon materials with large specific surface area and excellent electrical conductivity. These include graphene [19], mesoporous carbon [27], and Super P [24], which trap polysulfides through physical adsorption. However, the weak affinities of these carbon materials with polar polysulfides make it difficult to maintain the required long cycle stability of Li–S batteries, especially when using cathodes with high area sulfur loadings. Second, polar materials including metal sulfides [20, 32], metal nitrides [33, 34], and metal phosphides [35, 36] can be used. These materials exhibit excellent electrocatalytic performance for polysulfides and also strong chemical adsorption for these species. Further, they have been proven to be effective in limiting polysulfide shuttling and improving the redox reaction kinetics of polysulfides. However, to obtain high-performance Li–S batteries, the functionalized separator must be able to solve more than these two problems.

In the second stage of the discharge process, soluble polysulfides are further transformed into solid Li_2_S [7]. This electrochemical process contributes to three-quarters of the theoretical capacity of Li–S batteries [11]. However, the insulation and insolubility of the discharge product, Li_2_S, usually result in its aggregation [7, 11]. The cores of these Li_2_S aggregates become “dead sulfur” in the charging process because of the loss of electrical contact with the conductive network, leading to sluggish Li_2_S oxidation kinetics and the irreversible loss of active sulfur [7, 10, 11]. Therefore, regulating the uniform deposition of Li_2_S in the discharge process is crucial to improving the reversible capacity and cycling stability of Li–S batteries. Generally, the distribution of Li_2_S precipitates deposited on the matrix depends on the electron transfer capability of the matrix, the binding energy between Li_2_S and the matrix, and the distribution of active Li_2_S binding sites [7, 10–12, 15, 16, 25, 35–44]. Therefore, it is expected that Li–S batteries with separators functionalized with a material that strongly chemisorbs polysulfides, and exhibits excellent electrocatalytic performance for polysulfides and the capability to regulate the uniform nucleation and growth of Li_2_S could achieve excellent electrochemical performance.

**Fig. 1:**
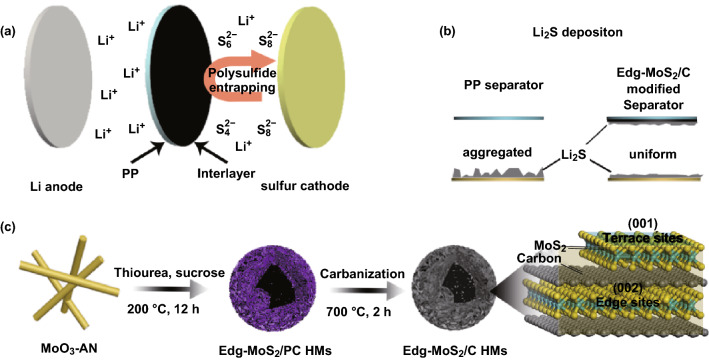
Schematic illustration of the synthesis and application of the Edg-MoS_2_/C@PP separators in Li–S batteries. **a** Schematic of polysulfides entrapped in the Li–S battery using the Edg-MoS_2_/C@PP separator. **b** Schematic of Li_2_S deposition in the Edg-MoS_2_/C@PP and PP cells. **c** Synthesis route of the Edg-MoS_2_/C HMs and schematic of the edge and terrace sites of the Edg-MoS_2_/C HMs

In this study, we first designed and fabricated hollow, edge-rich MoS_2_/C microsphere (Edg-MoS_2_/C HMs) functionalized separators to regulate the uniform deposition of Li_2_S in Li–S batteries. The Edg-MoS_2_/C HMs were obtained by the facile hydrothermal treatment of MoO_3_–aniline (MoO_3_–AN) nanowires with thiourea and sucrose and a subsequent carbonization process. These microspheres consist of uniformly distributed MoS_2_/C nanosheets that are rich in edge sites and a carbon network. The liquid-phase lithium polysulfides are effectively entrapped by MoS_2_ (Fig. 1a). Furthermore, the outstanding electrocatalytic performance of Edg-MoS_2_/C HMs accelerates the conversion kinetics of the polysulfides. Additionally, the carbon network facilitates electron transfer, and the many edge sites of the uniformly distributed MoS_2_/C nanosheets provide abundant, strong Li_2_S binding sites. As a result, the uniform nucleation and growth of Li_2_S on the matrix was realized (Fig. 1b). Moreover, the hollow structure and the ultrathin edge-rich MoS_2_/C nanosheets of the Edg-MoS_2_/C HMs increase the utilization of active edge sites and, thus, reduce the weight of the interlayers and guarantee the excellent performance of the corresponding Li–S batteries. Thanks to these advantages, the Edg-MoS_2_/C HMs-functionalized separators consequently improve the specific capacity, rate performance, and cyclic stability of Li–S batteries, especially with high sulfur contents and high areal sulfur loadings of cathodes.

## Experimental Section

### Preparation of Edg-MoS_2_/C HMs and Carbon Network

The MoO_3_–AN nanowires were prepared according to a previously reported method [45]. In a typical procedure, 100 mg of as-obtained MoO_3_–AN, 1.0 g thiourea, and 300 mg sucrose were added to 60 mL deionized water. Then, the mixture was ultrasonicated for 1 h. Subsequently, the obtained suspension was transferred to a 100-mL Teflon-lined autoclave, which was heated to 200 °C for 12 h in an oven. The resultant black products were collected by vacuum filtration, washed with deionized water and ethanol several times, and then dried in a vacuum oven at 60 °C for 12 h. The obtained product was the Edg-MoS_2_/partially carbonized carbon hollow microspheres (Edg-MoS_2_/PC HMs). The Edg-MoS_2_/C HMs were obtained after annealing in a tube furnace at 700 °C for 2 h under N_2_ flow. Edg-MoS_2_/C400 HMs and MoS_2_ microflowers (MFs) were obtained using the same procedures with or without the addition of 400 mg sucrose, respectively. In addition, the MoS_2_ in the Edg-MoS_2_/C HMs was removed with aqua regia, leaving only the carbon network (CN).

### Preparation of Edg-MoS_2_/C@PP and CN@PP Separators

The Edg-MoS_2_/C HMs (or CN), Super P (super conductive carbon black), and poly(vinylidene fluoride) binder (6:3:1 in mass ratio) were stirred in *N*-methyl-2-pyrrolidone (NMP) to form a uniform slurry, which was then coated on a Celgard 2400 polypropylene (PP) membrane. After drying in a vacuum oven at 60 °C overnight, the Edg-MoS_2_/C@PP and CN@PP separators were obtained and punched into disks with diameters of 19 mm. The mass loadings of the coating materials on the Edg-MoS_2_/C@PP and CN@PP separators were controlled to be approximately 0.34 mg cm^−2^.

### Preparation of the Sulfur Cathode

Typically, sublimed sulfur and carbon nanotubes (CNTs, 4:1 by mass) were ground uniformly together and sealed in a 50-mL reaction kettle. The mixture was then heated at 155 °C for 10 h. The obtained product, CNT/S (approximately 80 wt%), carbon black (10 wt%), and LA133 binder (10 wt%) were stirred in an aqueous *n*-propanol solution. The slurry was coated onto carbon-coated aluminum foil and dried at 60 °C overnight. Then, the sulfur electrode was obtained and punched into disks with diameters of 12 mm. The average sulfur loading mass on the electrodes was controlled to be 1.7 mg cm^−2^. Higher sulfur loadings of 3.5 and 6.1 mg cm^−2^ were also prepared.

### Lithium Polysulfide Adsorption Tests

The lithium polysulfide adsorption tests were conducted by adding an equivalent amount (10 mg) of Edg-MoS_2_/C HMs and CN to the lithium polysulfide (Li_2_S_6_) solution and holding for 6 h. The lithium polysulfide (Li_2_S_6_) solution was prepared by dissolving stoichiometric amounts of sulfur and lithium sulfide (Li_2_S) in a molar ratio of 5:1 in a 1,2-dimethoxyethane/1,3-dioxolane (DME/DOL) mixture (1:1 by volume).

### Assembly of Symmetric Cells and Kinetic Evaluation of Polysulfide Conversion

Corresponding electrodes were fabricated without sulfur. The active material (Edg-MoS_2_/C HMs and CN) and poly(vinylidene fluoride) (PVDF) in a mass ratio of 6:1 were dispersed in NMP by vigorous stirring to form uniform slurry, which was subsequently overlaid on Al foils with an areal loading of approximately 0.31 mg cm^−2^. Two identical electrodes were used as the working and the counter electrodes. They were assembled into typical 2032-type coin cells with a PP membrane as the separator and 40.0 μL Li_2_S_6_ electrolyte (containing 0.5 mol L^−1^ Li_2_S_6_ and 1.0 mol L^−1^ lithium bis(trifluoromethanesulfonyl)imide (LiTFSI) in DOL/DME solution with a volume ratio of 1:1). The cyclic voltammetry (CV) curves of the symmetric cells were measured at scan rates of 50–2000 mV s^−1^ with a voltage range between − 1.0 and 1.0 V.

### Fabrication of Li–S Cells and Electrochemical Measurements

The electrochemical performances of the Li–S cells were determined in CR2032 coin cells with CNT/S electrodes as the cathodes, functionalized PP films as the separators, and lithium foil as the anodes. The electrolyte contained 1 M LiTFSI in DOL and DME binary solvents (1:1 by volume) with 2 wt% LiNO_3_. The ratio of added electrolyte to sulfur in the coin cells was 12 µL mg^−1^ sulfur. The amounts of electrolyte added to the cell with sulfur areal loadings of 1.7, 3.5, and 6.1 mg cm^−2^ were 23, 47, and 83 μL, respectively. The galvanostatic charge–discharge tests were carried out using a battery test system (CT2001C, LAND) in a voltage range of 1.8–2.7 V. CV measurements and electrochemical impedance spectroscopy (EIS) were conducted using an electrochemical workstation (Chenhua CHI 600). CV measurements were carried out from 1.7 to 2.8 V, and all electrochemical tests were performed at room temperature.

### Characterization

The crystal structures of the samples were determined using powder X-ray diffractometry (XRD) measurements on a Bruker/D8 Focus diffractometer (Germany). Cu Kα X-rays (*λ* = 1.5405 Å) were generated at 40.0 kV and 40 mA, and reflections were recorded in the 2*θ* range of 5°–80° at a scan speed of 6° min^−1^. N_2_ adsorption–deposition isotherms were collected with a Micromeritics TriStar II 3020 system mode at 77 K. All the samples were degassed at 100 °C for 15 h under flowing N_2_ before measurement. Field-emission scanning electron microscopy (FE-SEM) images and transmission electron microscopy (TEM) images were obtained using a Hitachi S4800 electron microscope (Japan) and a JEOL-2100F electron microscope (Japan), respectively. X-ray photoelectron spectroscopy (XPS) measurements were conducted on a Thermo Escalab 250 system. Raman spectra were measured with an excitation laser wavelength of 514.5 nm at room temperature using a LabRAM HR. Thermogravimetric (TG) analyses of the Edg-MoS_2_/C HMs and the CNT/S composite were performed in a PerkinElmer (TA Instruments) up to 650 °C at a heating rate of 10 °C min^−1^ in air and N_2_, respectively.

## Results and Discussion

As shown in Fig. 1c, the Edg-MoS_2_/C HMs were synthesized by the facile hydrothermal treatment of MoO_3_–AN nanowires with thiourea and sucrose and a subsequent carbonization process. During the hydrothermal treatment process, MoO_3_–AN nanowires were first transformed into solid microspheres (Fig. S1b) and then gradually transformed into hollow microspheres (Fig. S1b–g) by the Kirkendall effect, yielding Edg-MoS_2_/PC HMs (Fig. S1 g) [46–48]. Subsequently, carbonization was conducted to enhance the degree of graphitization of the carbon network in the Edg-MoS_2_/C HMs. In addition, MoS_2_ in the Edg-MoS_2_/C HMs was removed using aqua regia, and the CN alone was, thus, obtained.

**Fig. 2:**
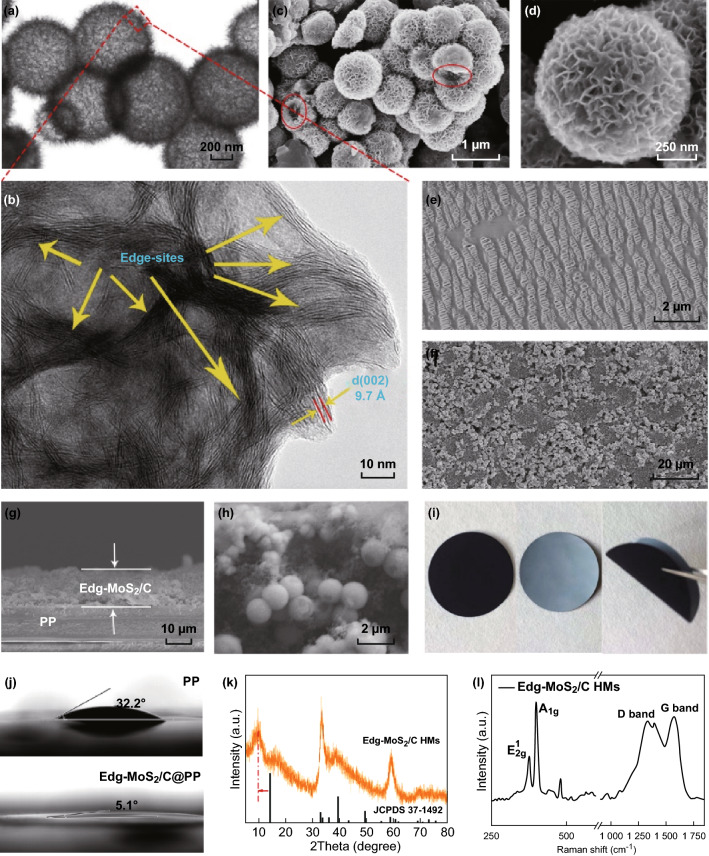
Characterization of the Edg-MoS_2_/C HMs and Edg-MoS_2_/C@PP separators. TEM images **a** at low and **b** high magnifications, and SEM images **c** at low and **d** high magnifications of the Edg-MoS_2_/C HMs. Top-down SEM images of **e** the pristine PP separator and **f** Edg-MoS_2_/C@PP separator. **g** Low- and **h** high-magnification cross-sectional SEM images of the Edg-MoS_2_/C@PP separator. **i** Optical photographs of Edg-MoS_2_/C@PP separator. **j** Contact angle between electrolyte and PP separator (top), electrolyte and Edg-MoS_2_/C@PP separator (bottom). **k** XRD pattern, **l** Raman spectrum of the Edg-MoS_2_/C HMs

The TEM image in Fig. 2a and the SEM images in Fig. 2c, d show the hollow structure of the Edg-MoS_2_/C HMs where a number of nanosheets are attached to the shells. High-magnification TEM images (Figs. 2b and S2) further reveal that the shells of the Edg-MoS_2_/C HMs are composed of edge-rich MoS_2_/C nanosheets and a carbon network. The interlayer distance of the MoS_2_ (002) crystalline planes was measured to be around 9.7 Å. As illustrated in the low-magnification TEM image (Fig. 2a), the average diameter of the Edg-MoS_2_/C HMs particles is a few hundreds of nanometers, and the average thickness of the edge-rich MoS_2_/C nanosheets is about 10 nm. The observed uniformly distributed edge sites in the MoS_2_/C nanosheets provide abundant Li_2_S deposition sites. The hollow structure and ultrathin edge-rich MoS_2_/C nanosheets increase the utilization of active edge sites, thus reducing the weight of the interlayers while ensuring excellent performance of Li–S batteries. The MoS_2_ content in the Edg-MoS_2_/C HMs was calculated to be about 73% (Fig. S6a). The powder XRD pattern (Fig. 2k) illustrates the well-defined characteristic peaks of hexagonal MoS_2_, except for the (002) peak, which can be attributed to the expansion of MoS_2_ (002) crystal planes by the insertion of a carbon layer [49]. The presence of C–OH/C–O–Mo (285.3 eV) peaks in the C 1*s* XPS spectrum (Fig. S4c) and a C–O–Mo (532.7 eV) peak in the O 1*s* XPS spectrum (Fig. S4d) also confirm the chemical combination of hexagonal MoS_2_ and a carbon layer in the Edg-MoS_2_/C HMs [20, 50]. The Raman spectrum of the as-obtained Edg-MoS_2_/C HMs shows the existence of the carbon network and molybdenum disulfide (Fig. 2l) [49, 50]. In particular, the intensity of the D-band (corresponding to the *A*_1g_ vibration mode caused by defective *sp*^2^ carbon rings) is larger than that of the G-band (corresponding to the *E*_2g_^1^ vibration mode of *sp*^2^ carbon rings without defects), having a *I*_D_/*I*_G_ value of 0.99, which demonstrates the degree of graphitization of the carbon network [49, 50] and this means the enhancement of the overall conductivity of the Edg-MoS_2_/C HMs. The high specific surface area (28.8 m^2^ g^−1^) and uniform mesoporous distribution (approximately 4 nm) of the Edg-MoS_2_/C HMs (Fig. S5 and Table S1) are expected to provide abundant reaction sites of sulfur species in the charge–discharge processes [7].

On the basis of these results, Edg-MoS_2_/C HMs were employed to modify commercial PP separators for Li–S batteries. Unlike the macroporous structure of the PP separator (Fig. 2e), the Edg-MoS_2_/C interlayer was constructed of homogeneously distributed Edg-MoS_2_/C HMs with Super P (Fig. 2f–h), as shown in both the top-down and cross-sectional SEM images. In the Janus-structured Edg-MoS_2_/C-functionalized separator (Edg-MoS_2_/C@PP) (Fig. 2g), the Edg-MoS_2_/C interlayer side toward the cathode can be used to block the diffusion of polysulfides and improve the homogeneous deposition of Li_2_S, whereas the non-conductive PP side toward the anode prevents direct contact between the cathode and anode [7, 10, 11, 25]. The thickness of Edg-MoS_2_/C interlayer is as low as about 15 μm (Fig. 2g), and its areal weight density was calculated to be about 0.34 mg cm^−2^. Moreover, the Edg-MoS_2_/C@PP separator can be bent significantly (Fig. 2i), indicating its good mechanical stability. Compared to that between the electrolyte and the pristine PP, the much smaller 5.1° contact angle (Fig. 2j) between the electrolyte and Edg-MoS_2_/C@PP guarantees the enhanced electrolyte wettability of the Edg-MoS_2_/C interlayer.

**Fig. 3:**
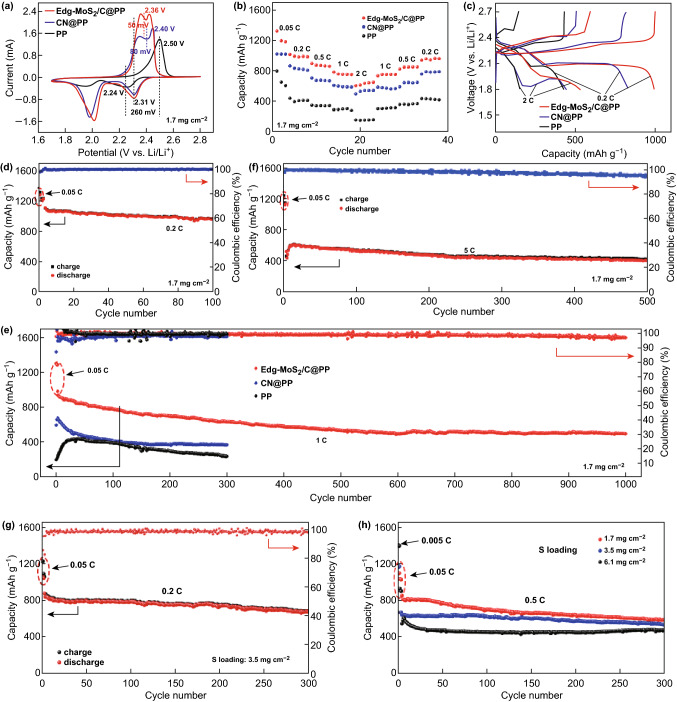
Electrochemical performances of the CNT/S cathodes with the Edg-MoS_2_/C@PP and PP separators. **a** Cyclic voltammogram profiles of the Edg-MoS_2_/C@PP, CN@PP, and PP cells at a scan rate of 0.1 mV s^−1^. **b** Rate capabilities and **c** corresponding galvanostatic discharge–charge profiles at 0.2 C and 2.0 C (1 C = 1675 mA g^−1^) of the Edg-MoS_2_/C@PP, CN@PP, and PP cells. Cycling performances of **d** the Edg-MoS_2_/C@PP cells at 0.2 C, **e** the Edg-MoS_2_/C@PP, CN@PP, and PP cells at 1.0 C, **f** the Edg-MoS_2_/C@PP cells at 5.0 C. The sulfur loadings of the CNT/S cathodes in **a–f** are 1.7 mg cm^−2^. Cycling performance of the Edg-MoS_2_/C@PP cells **g** at 0.2 C with a high sulfur loading of 3.5 mg cm^−2^, **h** at 0.5 C with sulfur loadings of 1.7, 3.5, and 6.1 mg cm^−2^, respectively. The weight of the interlayers in all cells is 0.34 mg cm^−2^

To validate the effects of the Edg-MoS_2_/C@PP separators on the electrochemical performances of Li–S cells, Li–S coin cells with the same CNT/S cathodes (sulfur areal loading of 1.7 mg cm^−2^) and Li foil anodes were assembled. Depending on the separator used, the assembled coin cells are designated Edg-MoS_2_/C@PP, CN@PP, and PP, respectively. As shown in Fig. 3a, CV measurements were used to explore the electrochemical processes occurring in the Li–S cells. Compared to those in the CV curves of CN@PP (1.98 and 2.31 V) and PP (1.94 and 2.24 V), the significantly enhanced current intensities and higher peak potentials (2.01 and 2.31 V) of the two cathodic peaks in the CV curve of Edg-MoS_2_/C@PP indicate the effective restriction and significantly improved polysulfide conversion kinetics [7, 9]. In the subsequent anodic scan, a lower potential (2.36 V) and larger peak area of the anodic peak in the CV curve of Edg-MoS_2_/C@PP were observed, and these originate from the accelerated dissolution and oxidation reaction kinetics of Li_2_S [9]. The much lower polarization potential (50 mV) of Edg-MoS_2_/C@PP (CN@PP: 90 mV and PP: 260 mV) reveals the impressive enhancement in the redox kinetics of the sulfur species [34].

To evaluate the role of the Edg-MoS_2_/C@PP separators on the redox reactions of sulfur species in Li–S cells further, the rate capabilities of Edg-MoS_2_/C@PP, CN@PP, and PP were measured. As shown in Fig. 3b, an initial discharge capacity of 1327 mAh g^−1^ for Edg-MoS_2_/C@PP was delivered at 0.05 C (1.0 C = 1675 mAh g^−1^). On increasing the current density to 0.2 C, 0.5 C, and 1.0 C, discharge capacities of 1014, 896, and 780 mAh g^−1^ were obtained, respectively. Even at a high current density of 2.0 C, the Edg-MoS_2_/C@PP still displayed a discharge capacity of 605 mAh g^−1^. On reducing the current densities to 1.0 C, 0.5 C, and 0.2 C, high reversible discharge capacities of 734, 823, and 934 mAh g^−1^ were maintained after the high rate test, corresponding to nearly 92% capacity recovery. In contrast, CN@PP displayed lower discharge capacities of 1020.5, 864.2, 705.4, 607.9, and 495.9 mAh g^−1^ at 0.05 C, 0.2 C, 0.5 C, 1.0 C, and 2.0 C, which was ascribed to the physical adsorption of polysulfides by the CN. PP exhibited obviously reduced capacities and rapid capacity degradation with increasing current density, exhibiting a much lower discharge capacity of 153 mAh g^−1^ at 2.0 C. The outstanding rate capability and capacity restoration of Edg-MoS_2_/C@PP can be attributed to the chemical affinity for polysulfides of Edg-MoS_2_/C HMs and the accelerated redox kinetics of the sulfur species in Edg-MoS_2_/C@PP. The galvanostatic charge–discharge plateaus of Edg-MoS_2_/C@PP, CN@PP, and PP show remarkable differences at current densities of 0.2 C and 2.0 C (Fig. 3c). Unlike those of CN@PP and PP, higher discharge plateaus and lower charge plateaus were exhibited in Edg-MoS_2_/C@PP. Even at 2.0 C, Edg-MoS_2_/C@PP still showed an apparent second discharge plateau, whereas CN@PP displayed a much lower second discharge plateau and PP had no second discharge plateau. These results demonstrate the rapid conversion kinetics of the polysulfides in the discharge process of Edg-MoS_2_/C@PP and that of Li_2_S in the charge process of Edg-MoS_2_/C@PP.

The improvement in the polysulfide shuttling through the Edg-MoS_2_/C HMs and the polysulfide and Li_2_S redox reaction kinetics were also investigated using long-term cycling tests of the Li–S cells. After three activation cycles at 0.05 C, Edg-MoS_2_/C@PP delivered an initial discharge capacity of 1106 mAh g^−1^ at 0.2 C (Fig. 3d). The discharge capacity of 957 mAh g^−1^ was retained in Edg-MoS_2_/C@PP after 100 cycles, accounting for 86.5% of the initial capacity, and the corresponding capacity decay was as low as 0.13% per cycle. This excellent behavior can probably be ascribed to the strong chemical affinity and excellent electrocatalytic performance toward polysulfides, as well as the enhanced Li_2_S conversion kinetics, of the Edg-MoS_2_/C HMs. The long-term cycling stability of Edg-MoS_2_/C@PP was further tested at 1.0 C for 1000 cycles. As shown in Fig. 3e, Edg-MoS_2_/C@PP displayed an initial discharge capacity of 935 mAh g^−1^ at 1.0 C after three cycles at 0.05 C. A reversible discharge capacity of 494 mAh g^−1^ with a coulombic efficiency above 97% was retained after 1000 cycles, demonstrating the long-term effectiveness of the Edg-MoS_2_/C HMs in promoting the redox reactions in Li–S cells. In contrast, the discharge capacities of CN@PP and PP were only 365.6 and 235 mAh g^−1^ at 1.0 C after 300 cycles, respectively, which could be ascribed to the sluggish conversion reaction and severe polysulfide shuttling, which would result in the low utilization of active sulfur, the corrosion of the Li anode, and the formation of lithium dendrites. This is confirmed by the SEM images of the Li anodes (Fig. S9), which were obtained by dissembling Li–S cells after 10 cycles at 1.0 C. The EIS measurements reveal the accelerated redox kinetics of the sulfur intermediates when using the Edg-MoS_2_/C HMs (Fig. S10). On increasing to 5.0 C, a discharge capacity of 602 mAh g^−1^ was obtained for Edg-MoS_2_/C@PP after three cycles at 0.05 C and 12 cycles at 5.0 C (Fig. 3f). After 500 continuous cycles, an excellent reversible capacity of 393 mAh g^−1^ remained, demonstrating the outstanding redox reaction kinetics of the Edg-MoS_2_/C HMs, even at high rates.

The mass ratio of MoS_2_ to C in the composite microspheres was adjusted to achieve the best performing Li–S battery. As illustrated in Fig. S15 and Table S2, Edg-MoS_2_/C400 HMs (44.4% MoS_2_) showed poor electrochemical performance because of the weak limiting effect on lithium polysulfides resulting from its low MoS_2_ content. Because the MoS_2_ MFs (98.1% MoS_2_) are solid structures with fewer active sites and poor conductivity arising from the ultra-low carbon content, this material also demonstrated poorer electrochemical performance than Edg-MoS_2_/C HMs (72.9% MoS_2_). Consequently, the Edg-MoS_2_/C HMs (72.9% MoS_2_) presented the best performance among these samples.

To explore the potential practical applications of Edg-MoS_2_/C@PP separators, the electrochemical performance of Li–S cells with higher sulfur areal loadings were tested. CNT/S cathodes with sulfur loadings of 1.7, 3.5, and 6.1 mg cm^−2^ were fabricated, and Li–S coin cells were assembled using the corresponding Edg-MoS_2_/C@PP separators, denoted CNT/S-1.7, CNT/S-3.5, and CNT/S-6.1, respectively. After three-cycle activation at 0.05 C, CNT/S-3.5 delivered an initial discharge capacity of 839 mAh g^−1^ (Fig. 3g) and a high capacity of 677 mAh g^−1^ with a coulombic efficiency of 98.6% after 300 cycles at 0.2 C, thus displaying a high capacity retention of 80.7% and a low capacity decay of 0.064% per cycle. Even at a high current density of 0.5 C, high discharge capacities of 594, 539, and 478 mAh g^−1^ (corresponding to areal capacities of 1.01, 1.89, and 2.92 mAh cm^−2^, respectively) were obtained for CNT/S-1.7, CNT/S-3.5, and CNT/S-6.1 after 300 cycles, respectively (Figs. 3h and S11), confirming the effective restriction of polysulfides and the enhancement in the reaction kinetics induced by the Edg-MoS_2_/C HMs, even in Li–S cells with high sulfur mass loadings and operated at a high rate. These results demonstrate that the Edg-MoS_2_/C@PP separators have potential practical applications in Li–S batteries. The rate performances and cycle stabilities of Li–S cells with Edg-MoS_2_/C@PP separators are comparable to those of recently reported Li–S cells with functionalized separators, as summarized in Tables 1 and S3 [19, 20, 24, 27, 32, 33, 51–56]. As illustrated in Table 1, although some of the interlayers of the listed MoS_2_-based materials are relatively thin and light, the performances of cells with these interlayer-modified separators at high areal sulfur cathode loadings were not provided, and this information is very important for assessing possible practical applications of Li–S batteries. However, the cells containing Edg-MoS_2_/C HM-functionalized separators exhibited excellent performance compared to those of other Li–S batteries at high sulfur contents and high areal sulfur loadings of the cathodes.

**Table 1:** Systematic performance comparison between Edg-MoS_2_/C HMs and previously reported similar materials

Barriers	Interlayers mass loading (mg cm^−2^)	Thickness of interlayers (μm)	Sulfur mass loading (mg cm^−2^)	Cathode (sulfur content)	Electrochemical performance				References
					Rate capacity (C)	Initial capacity (mAh g^−1^)	Cycles	Residual capacity (mAh g^−1^)/decay rate (%)	
MoS_2_/CNT	0.25	2	1.4	50	0.2	1205	200	770/0.18	[54]
					0.5	1237	500	648/0.061	
MoS_2_/graphene	0.5	60	0.8-1.2	60	0.12	1642	100	720	[55]
					0.3	–	200	689	
					0.6	840	200	620	
MoS_2_	–	0.35	–	65	0.5	808	600	401/0.083	[32]
rGO@MoS_2_	~ 0.24	~ 8	3.64	70	0.2	945	90	600/0.4	[20]
			1.8–2.0		0.2/1	1121/877	200/500	671/0.2 368/0.116	
MoS_2_-NPs	1.0	~ 25	4.0	70	0.2	983	150	525/0.34	[56]
Edg-MoS_2_/C HMs	0.34	15	1.7	64	0.2/1/5	1106/935/602	100/1000/500	957/0.13 494/0.047 393/0.069	This work
			3.5	64	0.2/0.5	839/653	300/300	677/0.064 539/0.058	
			6.1	64	0.5	554	300/300	478/0.046	

**Fig. 4:**
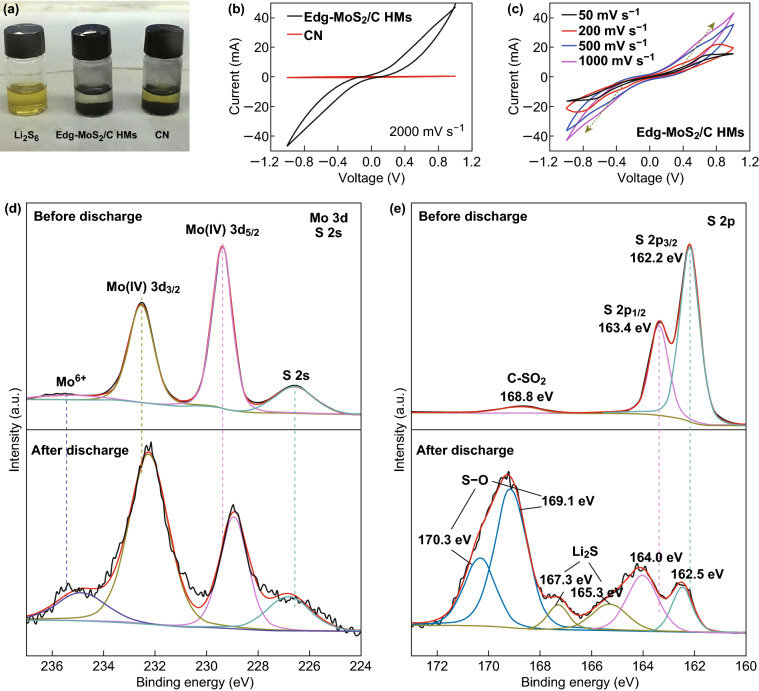
Investigation of the mechanism of interaction between the Edg-MoS_2_/C HMs and sulfur species during the redox reactions of the Li–S cells. **a** Visual adsorption tests of Li_2_S_6_ by pristine Edg-MoS_2_/C HMs and CN. **b** CV curves of symmetric dummy cells employing Edg-MoS_2_/C HMs and CN electrodes at a high scan rate of 2000 mV s^−1^. **c** CV curves of the Edg-MoS_2_/C HMs symmetric cells at different scan rates. **d** The Mo 3*d*, S 2*s*, and **e** S 2*p* spectrum of the Edg-MoS_2_/C-functionalized separator before and after the first 100% discharge at 0.05 C

To investigate the mechanism of interaction between the Edg-MoS_2_/C HMs and the sulfur species during the redox reactions of the Li–S cells, visual adsorption tests on Edg-MoS_2_/C HMs and CN of the same weight and lithium polysulfide content were carried out. After static adsorption for 6 h, the Li_2_S_6_ solution (Fig. 4a) with Edg-MoS_2_/C HMs was discolored, whereas the color of the Li_2_S_6_ solution with CN had not changed, indicating the strong chemical absorption of the soluble polysulfides by the Edg-MoS_2_/C HMs [7, 57]. Thus, the Edg-MoS_2_/C HMs can effectively prevent polysulfides from dissolving into the electrolyte, increasing the utilization of active sulfur and providing the preconditions for further conversion reactions of polysulfides.

XPS measurements were employed to determine the chemical interaction between MoS_2_ and the polysulfides. The high-resolution Mo 3d XPS spectrum (Fig. S13a) contains three typical peaks at 232.6, 229.4, and 235.4 eV, corresponding to Mo 3*d*_3/2_, Mo 3*d*_5/2_, and the + 6 oxidation state of Mo, respectively [58]. The Mo 3*d*_3/2_ and Mo 3*d*_5/2_ peaks could be assigned to Mo^4+^ [58]. After the polysulfide adsorption experiments, all three peaks (especially the Mo^6+^ peak) were downshifted to lower energies, indicating the intense chemical interaction of exposed Mo sites with the sulfur of the polysulfides [58]. The S 2*s* peak at 226.6 eV (Fig. S13a), S 2*p*_1/2_ peak at 163.4 eV, and S 2*p*_3/2_ peak at 162.2 eV (Fig. S13b) correspond to S^2−^ in MoS_2_. The three peaks upshifted to 226.9, 163.6, and 162.5 eV, respectively, after adsorption, demonstrating the electron transfer from surface-exposed S atoms in MoS_2_ to Li atoms in polysulfides [58]. Notably, two new peaks at 164.0 and 164.8 eV can be attributed to the polysulfides [58], and a further two new peaks at 169.3 and 170.5 eV were assigned to the S–O bond in the oxidized sulfur reaction intermediates of polysulfides and MoS_2_, along with the reduction in the Mo oxidation state [58]. The chemical interaction between Edg-MoS_2_/C HMs and polysulfides significantly restricted the polysulfide shuttle effect.

The polysulfide conversion rate in the discharge process has a tremendous effect on the rate capabilities of the Li–S cells. To probe the effect of the Edg-MoS_2_/C HMs on the polysulfide conversion reaction, CV measurements of symmetric dummy cells containing the Li_2_S_6_ electrolyte were carried out [7, 25, 59]. In comparison with that of the CN electrode, the Edg-MoS_2_/C HMs electrode exhibits a much higher current density at a high scan rate of 2000 mV s^−1^ (Fig. 4b), suggesting enhanced conversion kinetics between the soluble polysulfides [7]. To explore the electrocatalytic performance of the Edg-MoS_2_/C HMs on the conversion of soluble polysulfides further, CV profiles at different scan rates were obtained (Fig. 4c). The current densities of the reduction and oxidation peaks were found to increase remarkably at scan rates from 50 to 1000 mV s^−1^, implying the significantly rapid redox reaction kinetics of the sulfur intermediates, even at high scan rates. Generally, at a fast scan rate, the redox reactions are severely constrained by the diffusion rate of the reacting substances. However, distinguishable redox peaks (Fig. 4c) could be still observed in the CV curve of the Edg-MoS_2_/C HM electrode at a high scan rate of 1000 mV s^−1^, indicating the excellent electrocatalytic effectiveness of the Edg-MoS_2_/C HMs toward soluble lithium polysulfides [7]. These effects mainly originate from the uniformly distributed edge sites resulting from the combination of MoS_2_ and a carbon network, which provides more active sites for electrocatalysis and accelerates the diffusion rate of electrons and Li^+^, thus boosting polysulfide conversion.

The chemical interaction between the edge sites in the Edg-MoS_2_/C HMs and Li_2_S was verified by the XPS results (Fig. 4d). Before discharge, the Mo 3*d*_3/2_, Mo 3*d*_5/2_, and Mo^6+^ peaks were located at 232.5, 229.4, and 235.6 eV in the Mo 3*d* spectrum, respectively. After the first 100% discharge at 0.05 C, the corresponding peaks downshifted to 232.2, 228.9, and 234.9 eV, respectively, which is attributed to the chemical affinity of the Mo atoms in the Edg-MoS_2_/C HMs and S atoms in the Li_2_S. In addition, the S 2*s* peak upshifted from 226.6 eV before discharge to 226.8 eV after the first 100% discharge at 0.05 C, which is due to the chemical interactions between the S atoms in the Edg-MoS_2_/C HMs and the Li atoms in the Li_2_S via chemical absorption. As shown in Fig. 4e, the S 2*p*_1/2_ peak at 163.4 eV and the S 2*p*_3/2_ peak at 162.2 eV in the S 2*p* spectrum correspond to S^2−^ in MoS_2_, and the corresponding peaks shifted to higher energies of 163.6 and 162.5 eV, respectively, after adsorption, demonstrating electron transfer from the surface-exposed S atoms in MoS_2_ to Li atoms in Li_2_S [58]. In addition, some new peaks emerged. The two peaks at 167.3 and 165.3 eV were attributed to Li_2_S [60], and a further two peaks at 169.1 and 170.3 eV were assigned to the S–O bonds in the oxidized sulfur species [58]. Therefore, a strong chemical interaction exists between Edg-MoS_2_/C HMs and Li_2_S, which facilitates the nucleation of Li_2_S on the Edg-MoS_2_/C HMs.

**Fig. 5:**
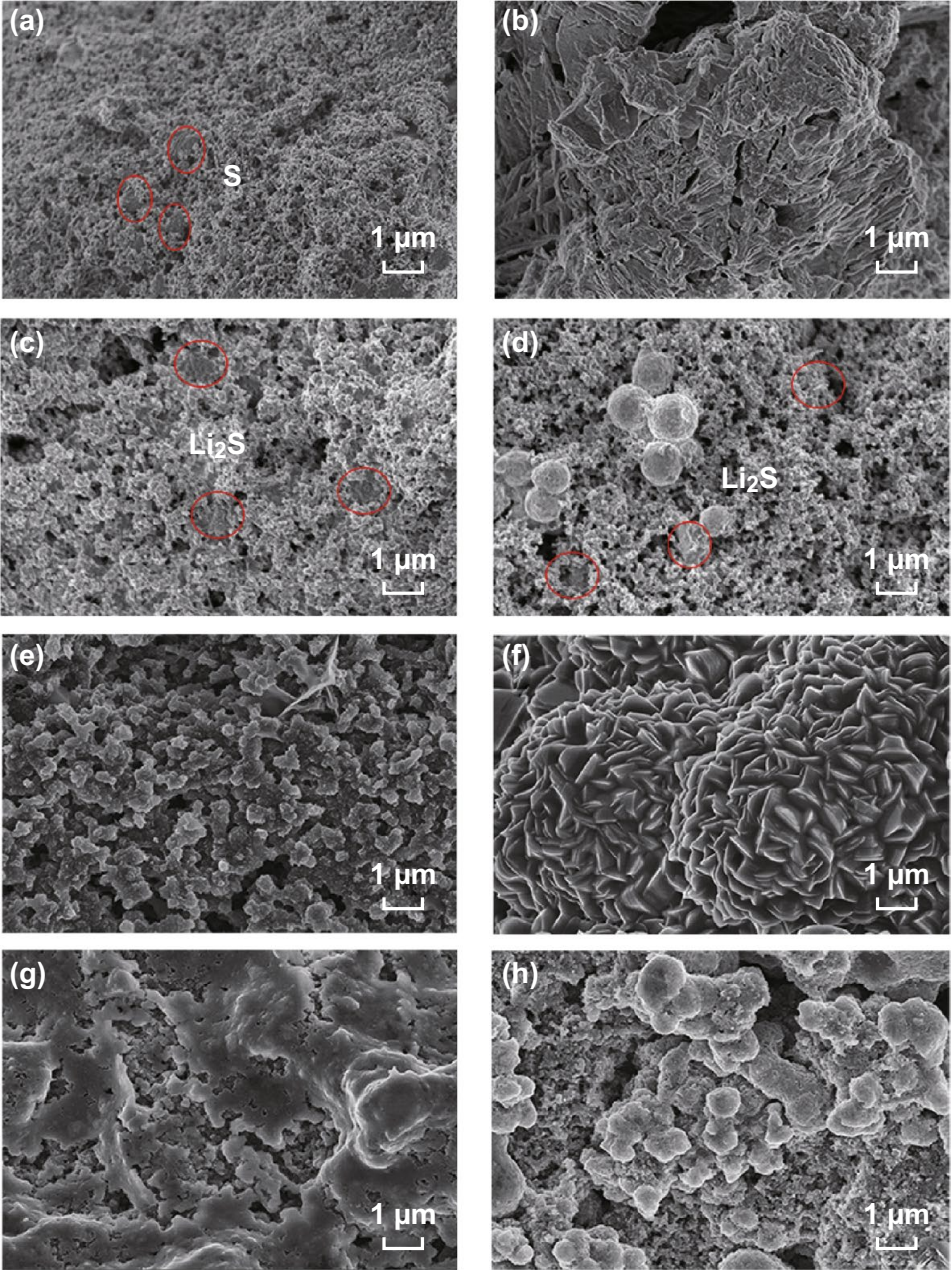
SEM images of Li_2_S and S deposition in the redox reactions of the Li–S cells. **a** SEM images of the pristine CNT/S-1.7 cathode. **b** SEM images of Li_2_S deposited on the CNT/S-1.7 cathode in the PP coin cell after the first 100% discharge at 0.05 C. SEM images of Li_2_S deposition on the CNT/S-1.7 cathode **c** and the separator **d** in the CN@PP coin cell after the first 100% discharge at 0.05 C. SEM images of Li_2_S deposition onto the CNT/S-1.7 cathode **e** and the separator **f** in the Edg-MoS_2_/C@PP coin cell after the first 100% discharge at 0.05 C. SEM images of the CNT/S-1.7 cathode **g** and the separator **h** in the Edg-MoS_2_/C@PP coin cell after the first 100% charge at 0.05 C

The rapid conversion kinetics of the lithium polysulfides and the strong chemical interactions between the Edg-MoS_2_/C HMs and Li_2_S create favorable conditions for the well-distributed nucleation and growth of Li_2_S in the second stage of the discharge process. To examine the deposition of Li_2_S on the matrix, the cell was disassembled after the first 100% discharge at 0.05 C. Impressively, the deep discharge product Li_2_S in Edg-MoS_2_/C@PP was deposited evenly along the surface of Edg-MoS_2_/C HMs in the Edg-MoS_2_/C interlayer and CNTs in the cathode (Fig. 5e, f), which is because the uniform edge sites of the Edg-MoS_2_/C HMs provided a large number of nucleation sites [40], and the strong chemical absorption and fast reduction capability for soluble polysulfides by Edg-MoS_2_/C HMs guided the uniform growth of Li_2_S. Sulfur particles were uniformly deposited on the cathode and the Edg-MoS_2_/C-modified separator (Fig. 5g, h). This is because the well-distributed Li_2_S precipitates in the discharge process are beneficial for the accelerated dissolution of Li_2_S, and the conversion of short-chain polysulfides to long-chain polysulfides is enhanced by the excellent electrocatalytic performance of the Edg-MoS_2_/C HMs. In contrast, it was found that the very large Li_2_S precipitates (Fig. 5b) (dozens of micrometers) were deposited on the surface of the cathode in PP, whereas the large Li_2_S precipitates measuring several micrometers (Fig. 5c, d) were deposited on the surfaces of the cathode and separator for the CN@PP cell. This was caused by the low density of nucleation sites in the cathodes with CN@PP and PP, as well as the sluggish polysulfide reduction kinetics. In addition, the physical adsorption of polysulfides by the CN interlayer occurred in CN@PP.

On the basis of our results, the well-distributed deposition of Li_2_S plays a significant role in the discharge and charge processes. Therefore, it is necessary to guide the uniform deposition of Li_2_S to achieve high-performance Li–S batteries. The binding affinity for polysulfides, electric conductivity, electrocatalytic performance of the matrix, and the density of Li_2_S binding sites, as well as their distribution on the matrix, are the key factors for controlling the deposition process of Li_2_S. In detail, (1) the hollow, edge-rich MoS_2_/C microspheres afford abundant chemical absorption sites for polysulfides, thus effectively restricting the polysulfide shuttle effect and providing the preconditions for the well-distributed deposition of Li_2_S. (2) The abundant edge sites exposed on the surface of Edg-MoS_2_/C HMs facilitate the nucleation and dissolution of Li_2_S, thus improving the reversibility or the phase conversion of the active species. (3) The intrinsic electrocatalytic performance of MoS_2_ and the enhanced electrical conductivity achieved because of the combination of MoS_2_ and the carbon network in the hollow, edge-rich MoS_2_/C microspheres, accelerated the conversion rate of sulfur intermediates, and guided the uniform growth of Li_2_S, which increased the utilization of active sulfur and improved the specific capacity and rate performance of the Li–S batteries. As a result, hollow, edge-rich MoS_2_/C microspheres can effectively regulate the uniform deposition of Li_2_S, resulting in high-performance Li–S batteries.

## Conclusion

In summary, hollow, edge-rich MoS_2_/C microspheres were successfully used to functionalize separators to regulate the uniform deposition of Li_2_S in lithium–sulfur batteries. We confirmed that Edg-MoS_2_/C HMs could restrict the polysulfide shuttle effect effectively and enhance the conversion kinetics of sulfur intermediates. More importantly, the uniform edge sites on the MoS_2_/C HMs provide abundant Li_2_S nucleation sites that guide the growth of Li_2_S, leading to well-distributed Li_2_S precipitates on the matrix. The cell containing a separator functionalized with hollow, edge-rich MoS_2_/C microspheres displayed an initial discharge capacity of 935 mAh g^−1^ at 1.0 C and maintained a capacity of 494 mAh g^−1^ after 1000 cycles at a sulfur loading of 1.7 mg cm^−2^. Impressively, at a high sulfur loading of 6.1 mg cm^−2^ and high rate of 0.5 C, the cell still delivered a high reversible discharge capacity of 478 mAh g^−1^ after 300 cycles. This work provides fresh views and solutions to the problems resulting from the complex phase conversion processes in energy storage systems.

## Electronic supplementary material

Below is the link to the electronic supplementary material.

Supplementary material 1 (PDF 1600 kb)
