# Supplementary material for: Battery Separators Functionalized with Edge-Rich MoS2/C Hollow Microspheres for the Uniform Deposition of Li2S in High-Performance Lithium–Sulfur Batteries
Source: Nanomicro Lett. 2019 May 24;11:43. doi: 10.1007/s40820-019-0275-z (PMC7770949; doi:10.1007/s40820-019-0275-z)
Supplement: Supplementary file 1 — Supplementary material 1 (PDF 1600 kb) [file 40820_2019_275_MOESM1_ESM.pdf]

Supporting Information for

Battery Separators Functionalized with Edge-Rich MoS_2/C Hollow Microspheres for the Uniform Deposition of Li_2S in High-Performance Lithium–Sulfur Batteries

Nan Zheng[1], Guangyu Jiang[1], Xiao Chen[1], Jiayi Mao[1], Nan Jiang[1], Yongsheng Li[1,] *

[1]Lab of Low-Dimensional Materials Chemistry, Key Laboratory for Ultrafine Materials of Ministry of Education, Shanghai Engineering Research Center of Hierarchical Nanomaterials, School of Materials Science and Engineering, East China University of Science and Technology, Shanghai 200237, People's Republic of China

*Corresponding author. E-mail: ysli@ecust.edu.cn (Yongsheng Li)

Supplementary Figures

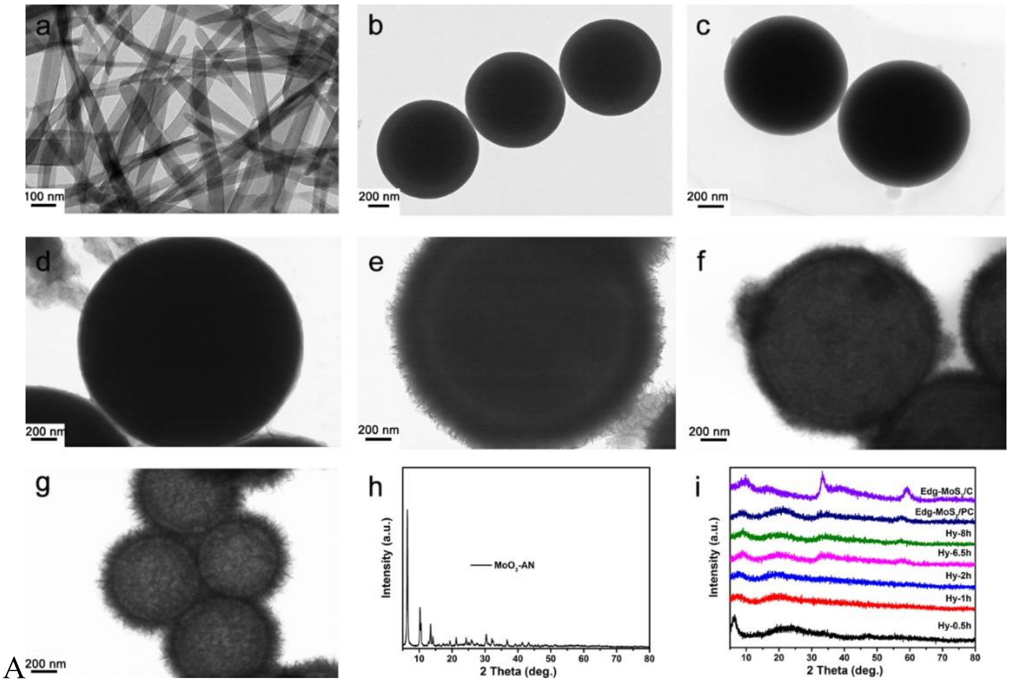

Fig. S1 Characterizations of the growth process of Edg-MoS_2/C HMs. **a** TEM image of MoO_3-AN. TEM images of the products at **b** 0.5 h, **c** 1 h, **d** 2 h, **e** 6.5 h, **f** 8 h, and **g** Edg-MoS_2/PC HMs in the hydrothermal process. **h** XRD patterns of MoO_3-AN, and **i** the growth process of Edg-MoS_2/C HMs in accordance with TEM images

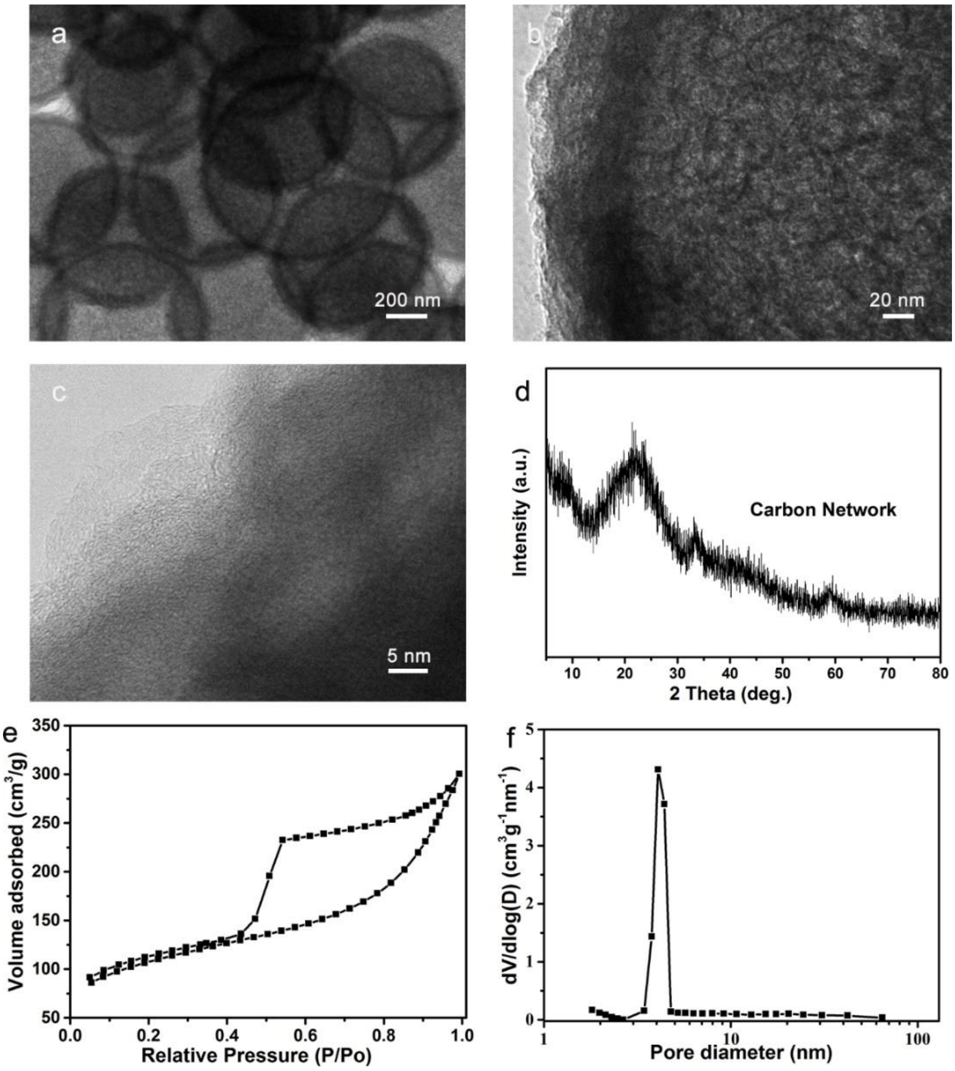

Fig. S2 a-c TEM images of the carbon network at different magnifications. **d** XRD patterns of the carbon network. **e** N_2 adsorption–desorption isotherms and **f** pore size distribution curves of the carbon network

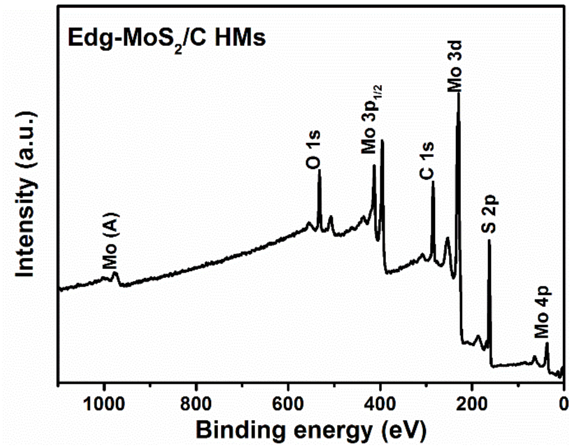

Fig. S3 XPS survey scan of the Edg-MoS_2/C HMs

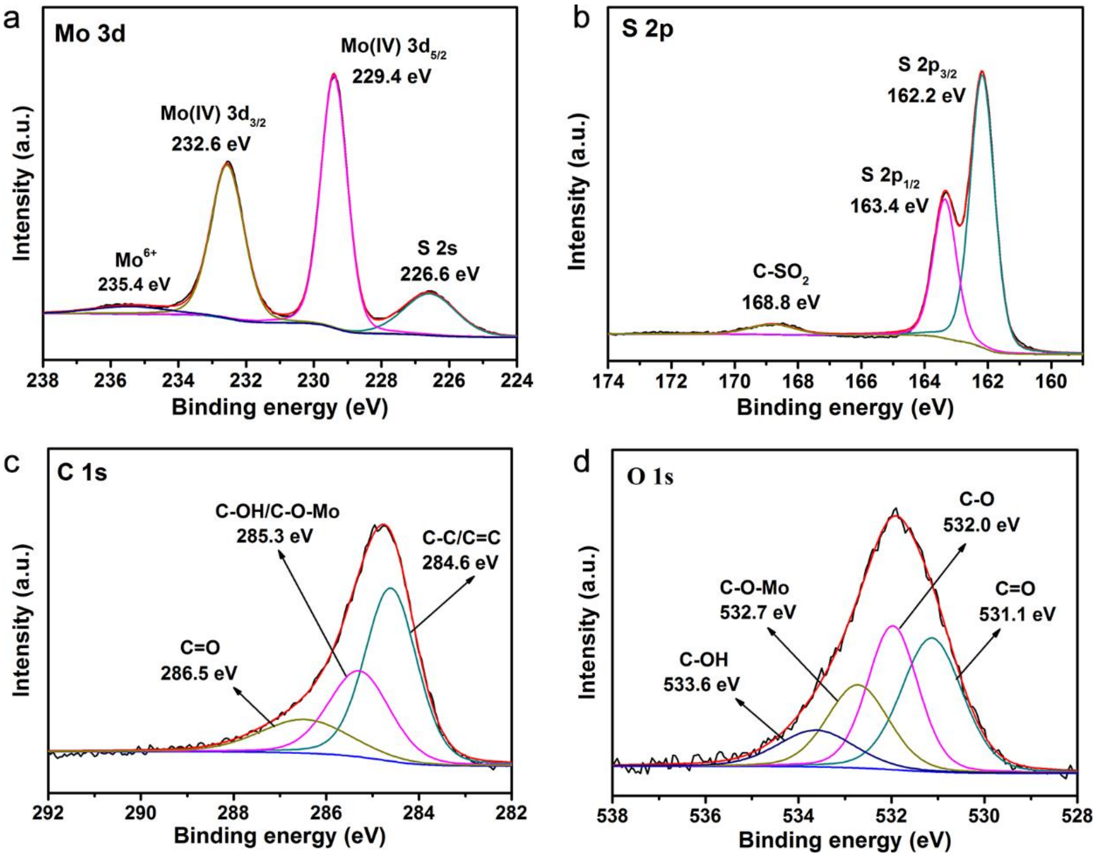

Fig. S4 a Mo 3d, **b** S 2p, **c** C 1s, and **d** O 1s XPS spectra of the Edg-MoS_2/C HMs

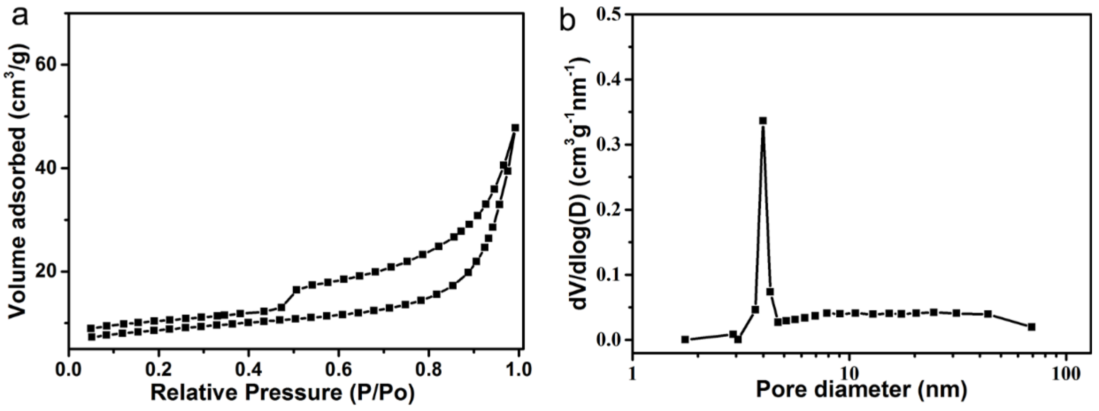

Fig. S5 a N_2 adsorption–desorption isotherms and **b** pore size distribution curves of the Edg-MoS_2/C HMs

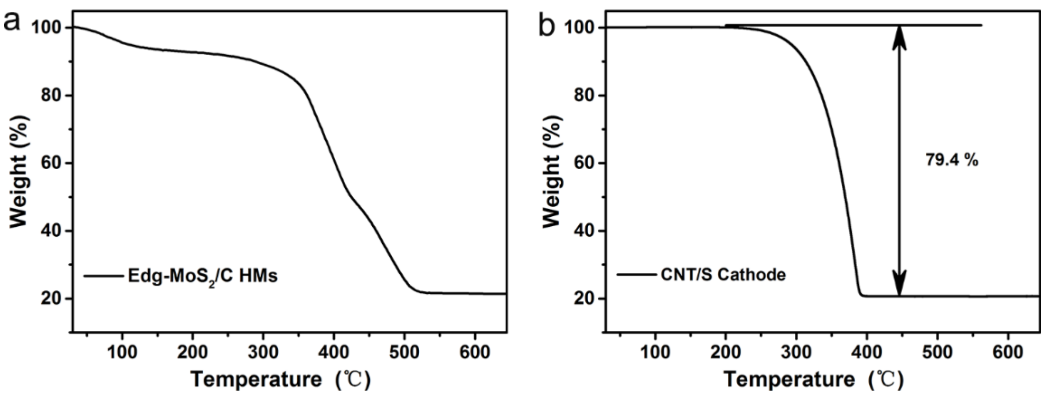

Fig. S6 TGA curves of **a** Edg-MoS_2/C HMs and **b** CNT/S composite

The Edg-MoS_2/C HMs were calcined in air and the residue is MoO_3. According to the chemical equation ($2MoS_2 + 7O_2 \rightarrow 2MoO_3 + 4SO_2$) and the obtained MoO_3 content, the content of MoS_2 in the Edg-MoS_2/C HMs could be calculated.

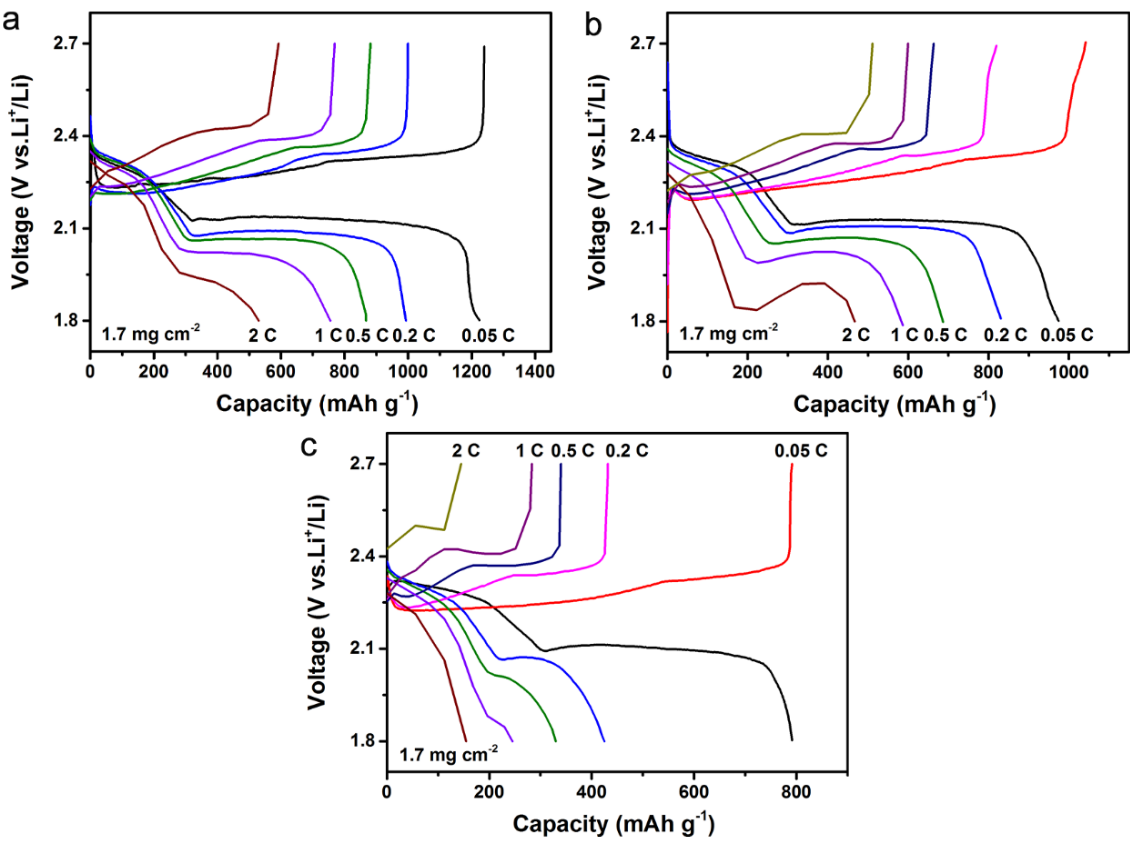

Fig. S7 Charge/discharge curves of **a** Edg-MoS_2/C@PP, **b** CN@PP, and **c** PP cells at different rates

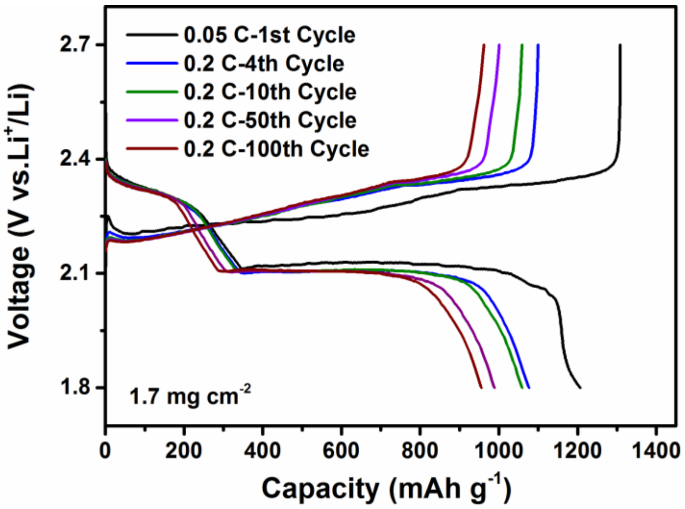

Fig. S8 Charge/discharge curves of the Edg-MoS_2/C@PP cells with high sulfur loading of 1.7 mg cm^{-2} at 0.2 C

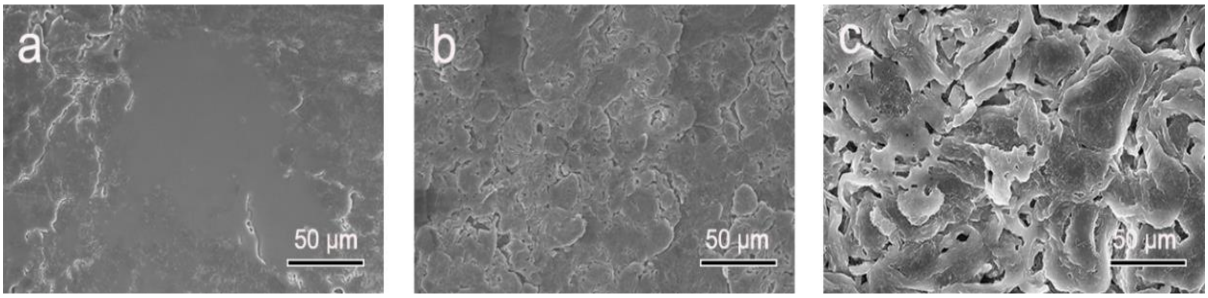

Fig. S9 SEM images of Li anodes by dissembling of **a** Edg-MoS_2/C@PP, **b** CN@PP, and **c** PP cells after 10 cycles at 1.0 C

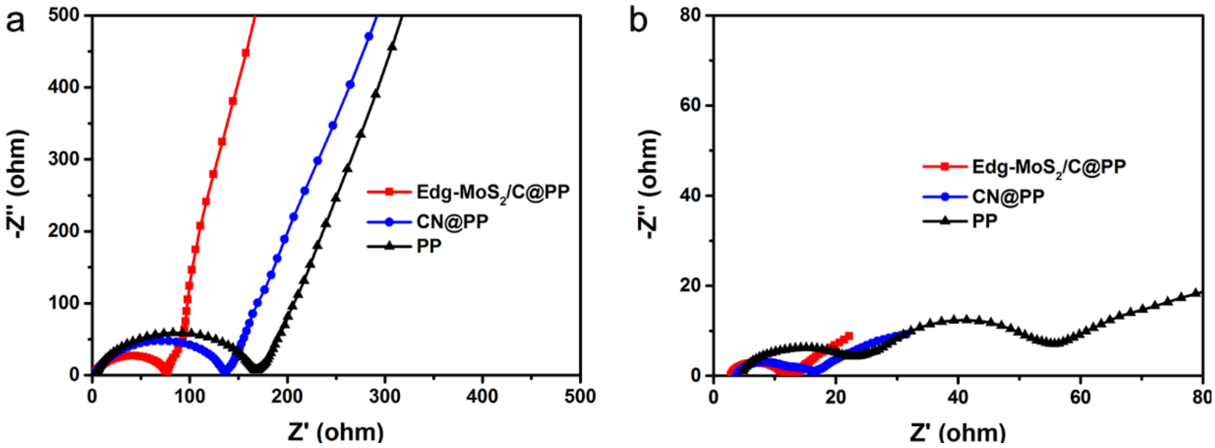

Fig. S10 EIS curves of the Edg-MoS_2/C@PP, CN@PP and PP cells **a** at fresh state and **b** after 10 cycles at 1.0 C

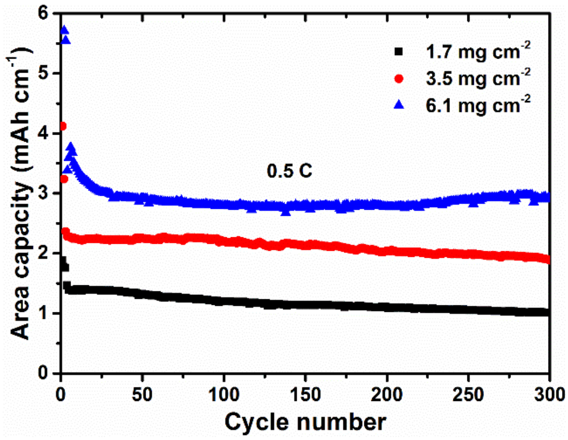

Fig. S11 The area capacity retentions of the Edg-MoS_2/C@PP cells with sulfur loadings of 1.7, 3.5, and 6.1 mg cm^{-2} at 0.5 C

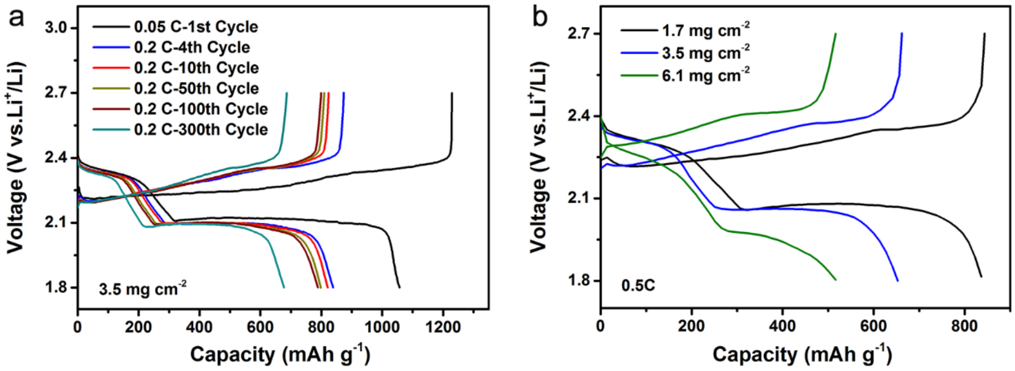

Fig. S12 Charge/discharge curves of the Edg-MoS_2/C@PP cells with **a** high sulfur loading of 3.5 mg cm^{-2} at 0.2 C, **b** sulfur loadings of 1.7, 3.5, and 6.1 mg cm^{-2} at 0.5 C

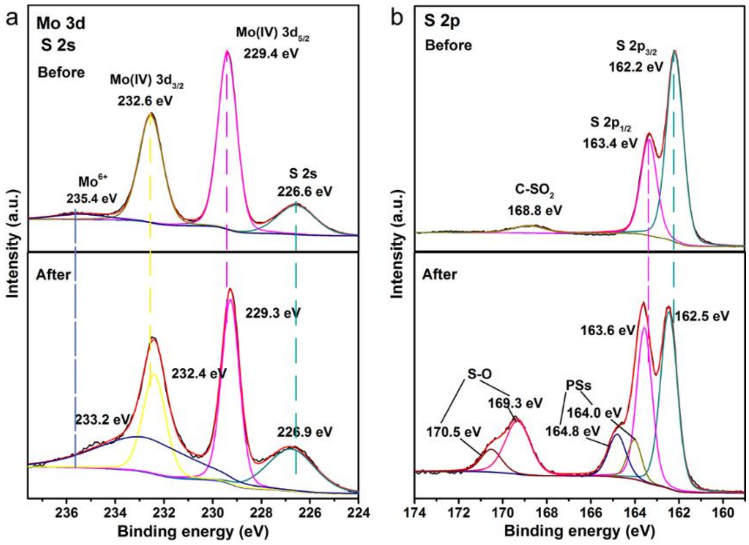

Fig. S13 XPS spectra of **a** Mo 3d and **b** S 2p of the Edg-MoS_2/C HMs before and after PSs adsorption

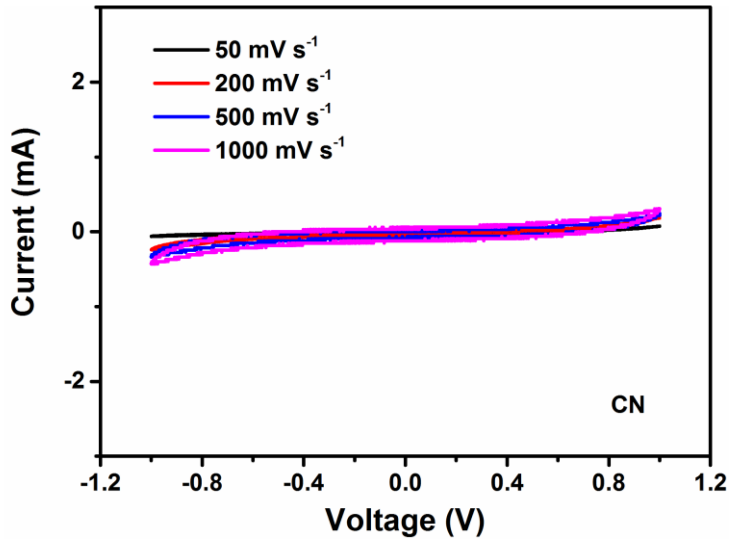

Fig. S14 CV curves of CN symmetric cells at different scan rates

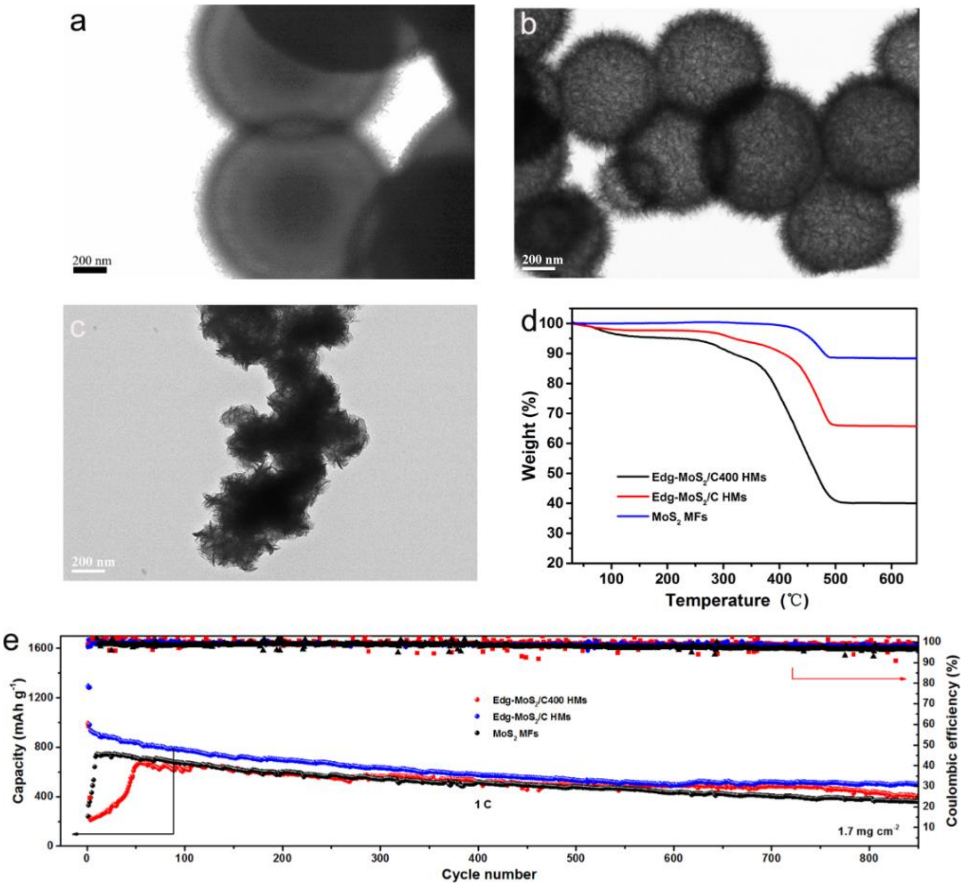

Fig. S15 TEM images of **a** Edg-MoS_2/C400 HMs, **b** Edg-MoS_2/C HMs, and **c** MoS_2 MFs. **d** TGA curves of the Edg-MoS_2/C400 HMs, Edg-MoS_2/C HMs, and MoS_2 MFs. **e** Cycling performances of the Edg-MoS_2/C400 HMs, Edg-MoS_2/C HMs and MoS_2 MFs cells at 1.0 C

Table S1 Structural Parameters for the Edg-MoS_2/C HMs and CN with N_2 sorption analysis

Sample	S_{BET} (m^2 g^{-1})	V (cm^3 g^{-1})	d (nm)
Edg-MoS_2/C HMs	28.8	0.07	4
CN	363.9	0.48	4

Table S2 TGA analysis results of the Edg-MoS_2/C400 HMs, Edg-MoS_2/C HMs, and MoS_2 MFs

Sample	Residual mass (%)	MoS_2 content (%)
Edg-MoS_2/C400 HMs	40.2	44.4
Edg-MoS_2/C HMs	66.1	72.9
MoS_2 MFs	88.7	98.1

Table S3 Comparison of electrochemical performance of Li-S batteries with different modified separators

Barriers	Interlayers mass loading (mg cm^{-2})	Thickness of interlayers (μm)	Sulfur mass loading (mg cm^{-2})	Cathode (Sulfur content)	Electrochemical performance				Refs.
					Rate Capacity (C)	Initial Capacity (mAh g^{-1})	Cycles	Residual capacity (mAh g^{-1}) /decay rate (%)	
Graphene	1.3	30	1.5~2.1	70	1	860	500	663/0.064	[S1]
Super P	0.61	60	0.70~1.0	60	1	/	200	721/N/A	[S2]
G-LTO	0.346	35	1.2	60	1	813	500	697/0.028	[S3]
Super P	0.38~0.52	10	1.0~1.4	63	0.35	1025	500	730/0.058	[S4]
Nafion–PP/PE/PP	0.7	/	0.53	50	1	781	500	469/0.08	[S5]
Mesoporous carbon	0.5	27	1.55	49	2	857	500	591/0.062	[S6]
NbC	0.9	10-	1.5	66.7	0.5	1082	150	872/0.13	[S7]
Edg-MoS_2/C	0.34	15	1.7	64	0.2/<br>1/<br>5	1106/<br>935/<br>602	100/<br>1000/<br>500	957/0.13<br>494/0.047<br>393/0.069	This work
			3.5	64	0.2/<br>0.5	839/<br>653	300/<br>300	677/0.064<br>539/0.058	
			6.1	64	0.5	554	300/<br>300	478/0.046	

Supplementary References

[S1]G. Zhou, L. Li, D.-W. Wang, X.-Y. Shan, S. Pei, F. Li, H.-M. Cheng, A flexible sulfur-graphene-polypropylene separator integrated electrode for advanced Li–S batteries. Adv. Mater. **27**(4), 641-647 (2014). https://doi.org/10.1002/adma.201404210

[S2]J. Zhu, Y. Ge, D. Kim, Y. Lu, C. Chen, M. Jiang, X. Zhang, A novel separator coated by carbon for achieving exceptional high performance lithium-sulfur batteries. Nano Energy **20**, 176-184 (2016). https://doi.org/10.1016/j.nanoen.2015.12.022

[S3]Y. Zhao, M. Liu, W. Lv, Y.-B. He, C. Wang, Q. Yun, B. Li, F. Kang, Q.-H. Yang, Dense coating of $Li_4Ti_5O_{12}$ and graphene mixture on the separator to produce long cycle life of lithium-sulfur battery. Nano Energy **30**, 1-8 (2016). https://doi.org/10.1016/j.nanoen.2016.09.030

[S4]H. Wang, W. Zhang, H. Liu, Z. Guo, A strategy for configuration of an integrated flexible sulfur cathode for high-performance lithium–sulfur batteries. Angew. Chem. Int. Ed. **55**(12), 3992-3996 (2016). https://doi.org/doi:10.1002/anie.201511673

[S5]J.-Q. Huang, Q. Zhang, H.-J. Peng, X.-Y. Liu, W.-Z. Qian, F. Wei, Ionic shield for polysulfides towards highly-stable lithium–sulfur batteries. Energy Environ. Sci. **7**(1), 347-353 (2014). https://doi.org/10.1039/C3EE42223B

[S6]J. Balach, T. Jaumann, M. Klose, S. Oswald, J. Eckert, L. Giebeler, Functional mesoporous carbon-coated separator for long-life, high-energy lithium–sulfur batteries. Adv. Funct. Mater. **25**(33), 5285-5291 (2015). https://doi.org/doi:10.1002/adfm.201502251

[S7]W. Cai, G. Li, K. Zhang, G. Xiao, C. Wang, K. Ye, Z. Chen, Y. Zhu, Y. Qian, Conductive nanocrystalline niobium carbide as high-efficiency polysulfides tamer for lithium-sulfur batteries. Adv. Funct. Mater. **28**(2), 1704865 (2018). https://doi.org/doi:10.1002/adfm.201704865
